# Supplementary material for: Thermostable virus portal proteins as reprogrammable adapters for solid-state nanopore sensors
Source: Nat Commun. 2018 Nov 7;9:4652. doi: 10.1038/s41467-018-07116-x (PMC6220183; doi:10.1038/s41467-018-07116-x)
Supplement: Supplementary file 1 — Supplementary Information [file 41467_2018_7116_MOESM1_ESM.pdf]

Supplementary Information

Biomolecule Sensing by Lipid-Free Anchoring of a Hydrophilic Thermostable Portal Adapter into Solid-State Nanopores

Benjamin Cressiot et al.

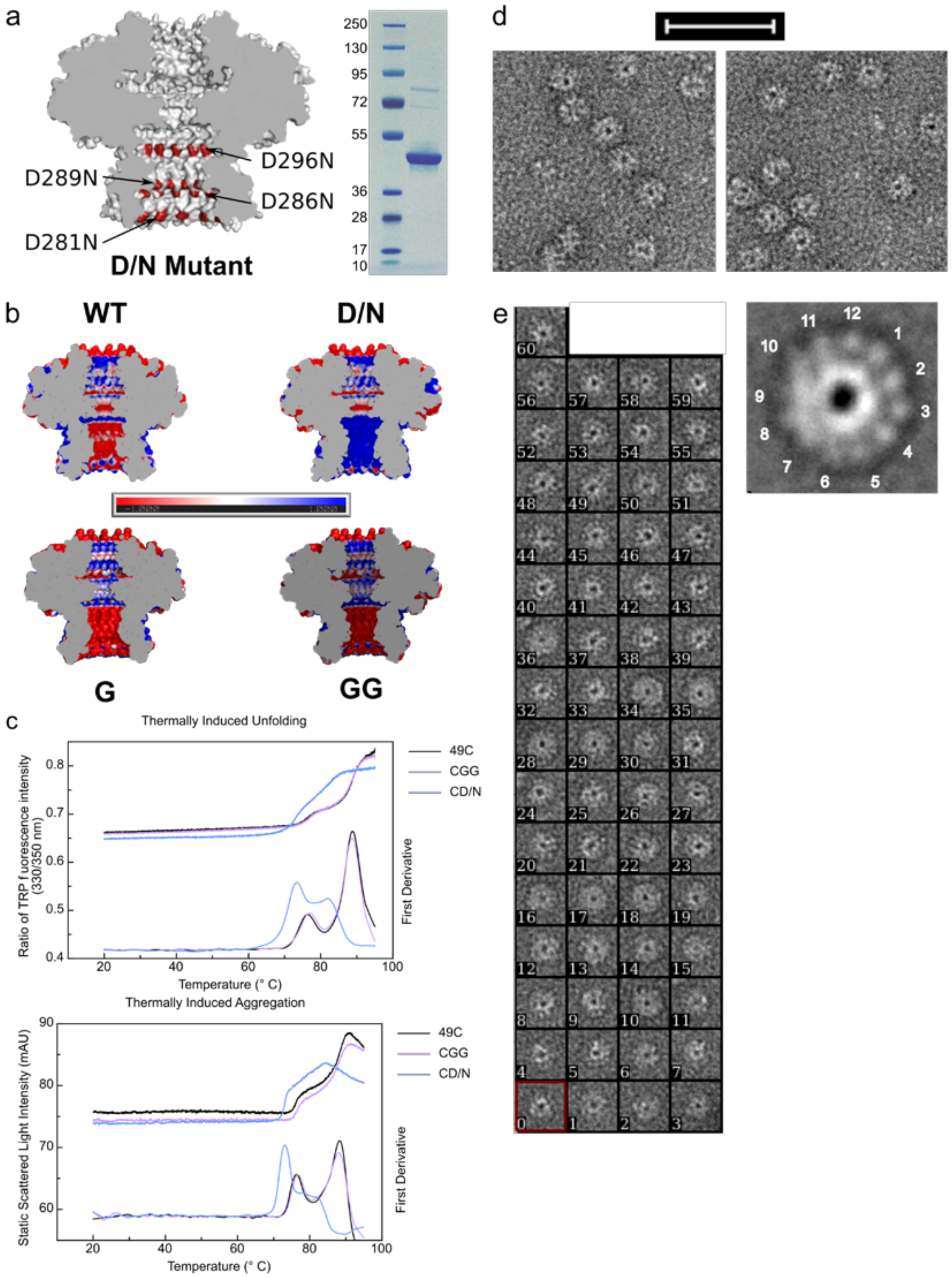

Supplementary Figure 1: Characterization of *G20c* Portal Protein.

a) Left: Four aspartic acid residues whose side chains project into the internal tunnel of the portal protein assembly were mutated to asparagine residues to create the D/N mutant. The mutant codons were introduced into the gene using a HiFi recombination assembly kit (New England Biolabs) to fuse a DNA fragment coding for amino acids 281-296 with the rest of the expression plasmid amplified by long PCR (Phusion, New England Biolabs). The dsDNA fragment

containing the mutated codons was assembled from contiguous overlapping complementary synthetic primers (Eurofins) and ligated with T4 ligase prior to gel purification and use in the HiFi assembly reaction. Creation of the mutant expression construct was confirmed by Sanger sequencing (GATC). Protein was expressed and purified as described previously[1]. Right: SDS-PAGE analysis of D/N mutant of the *G20c* portal protein compared with prestained protein standards (kDa; New England Biolabs). b) Electrostatic surface potentials (−1 (red) to +1 (blue) $kT\ e^{-1}$ represented by the scale bar) calculated using the Parse forcefield in PDB2PQR[2] and APBS[3] at 0.5 M NaCl pH 7, for the WT protein (PDB code: 4zjn) and V325G mutant (PDB code: 5ngd) and a models of the D/N mutant, created from 4zjn, and V325GI328 mutant, created from 5ngd, with Coot[4]. Images were prepared using PyMol (Schrödinger LLC). c) Thermal protein stability was defined by nanoDSF as unfolding (ratio of tryptophan fluorescence 330/350 nm; top) and aggregation (intensity of scattered light; bottom) in 20 mM Tris pH 7.5, 0.5 M NaCl on a Prometheus instrument (NanoTemper). d) Raw transmission electron microscope images of CD/N portal proteins negatively stained with 2% uranyl acetate imaged on an FEI T12 120 kV instrument at 98000 x magnification. Scale bar above the image is equivalent to 50 nm. e) 2D class average of CD/N portal protein showing the tunnel and the circular array of subunits. Images were taken at 98000 x magnification, ~80-100 $e^{-}\ A^{-2}$ and ~0.5 µM defocus. Images and particles were analyzed using EMAN2[5].

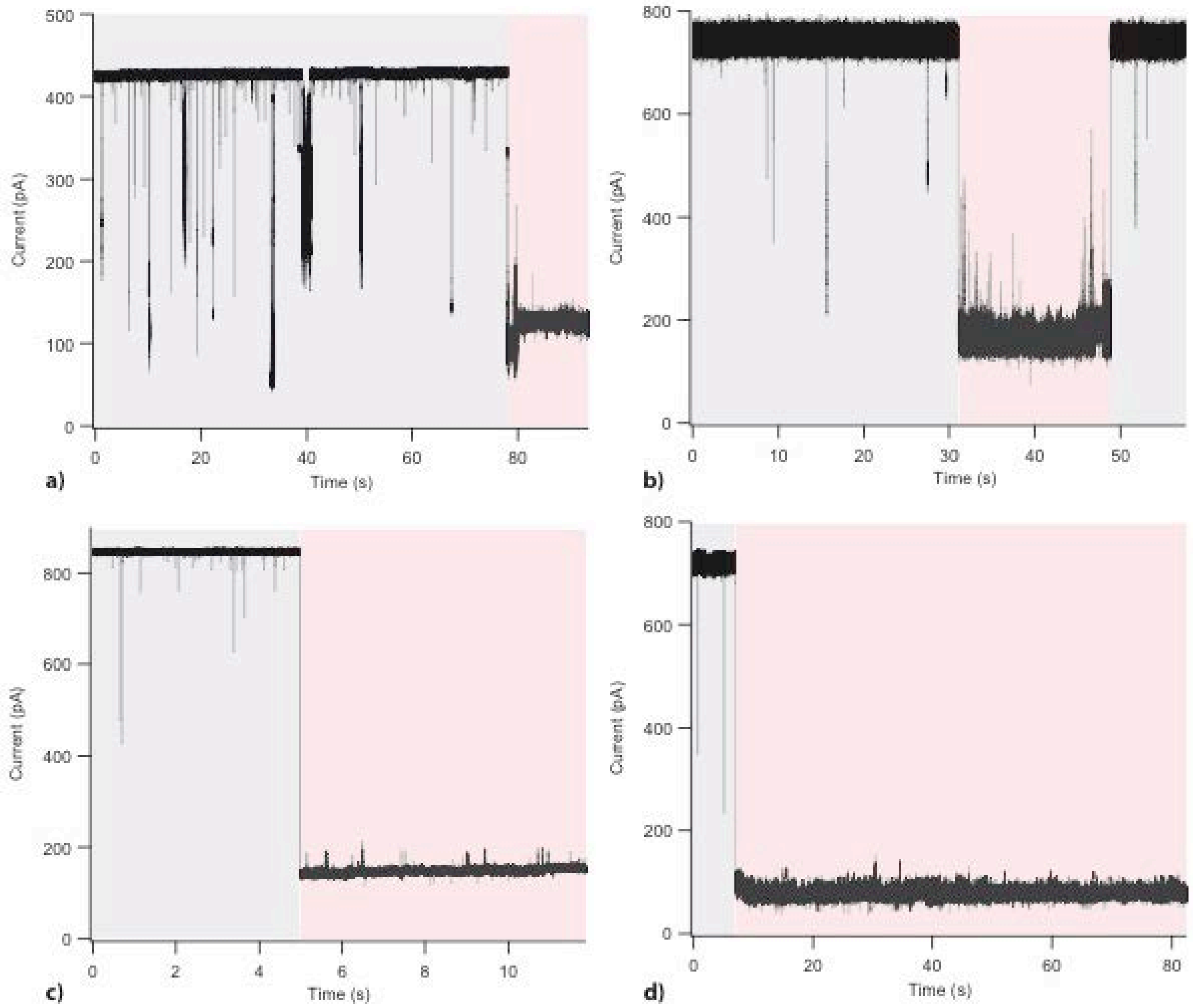

Supplementary Figure 2: Examples of portal protein CD/N insertions into solid-state nanopores. The traces show the recorded current of four independent solid-state nanopore before (grey) and after (red) insertion of a portal protein. The traces are recorded at +80 mV (a) and +100 mV (b, c, d) through pores of 5.4 nm diameter (a), 5.6 nm diameter (b, c), 5.5 nm diameter (d). Experiments were performed in 0.5 M NaCl, 20 mM Tris pH 7.5.

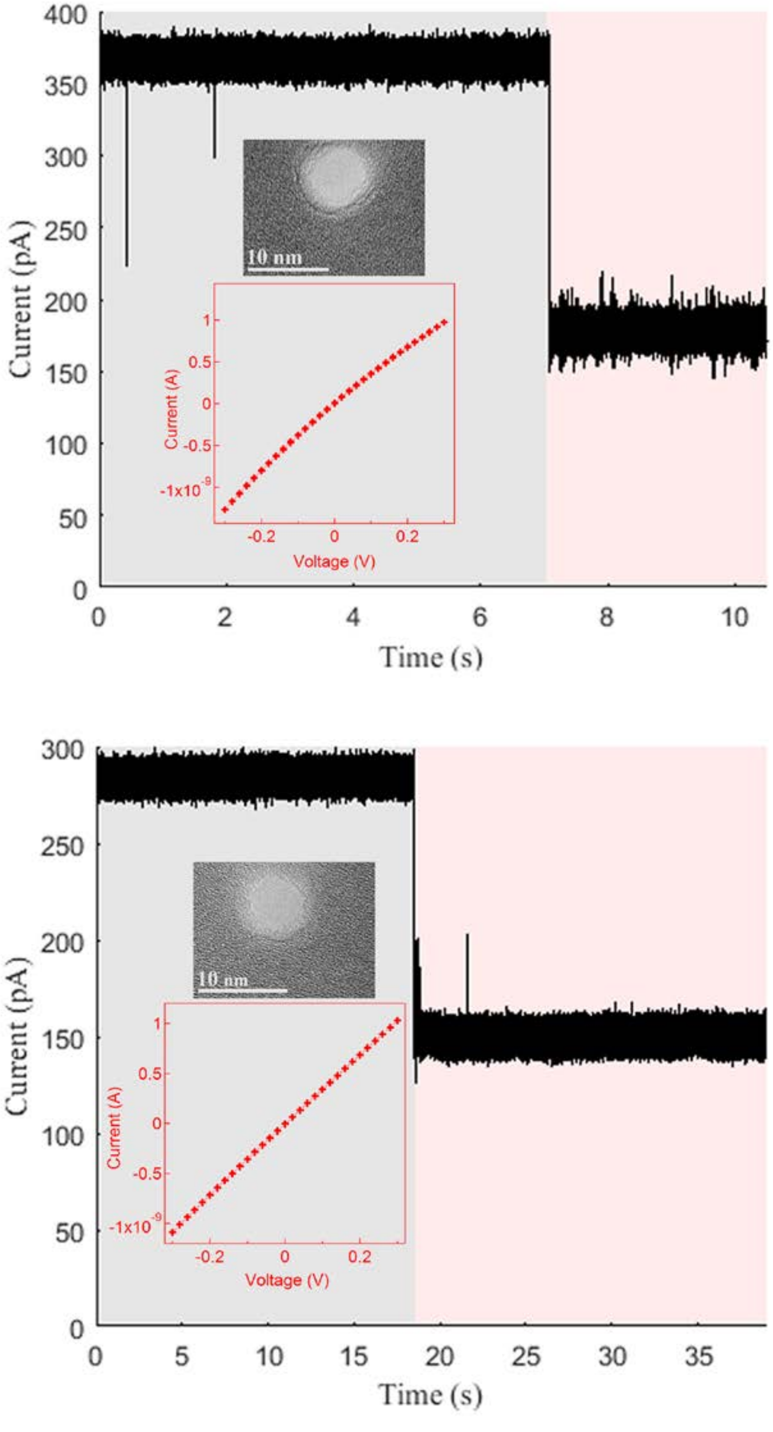

Supplementary Figure 3: Examples of portal protein CGG insertions into solid-state nanopores. Two examples of open solid-state pore current trace (grey) and hybrid pore current trace after CGG protein insertion (red). Experiments were conducted in 0.5 M NaCl, 20 mM Tris pH 7.5 and recorded at +100 mV with pore size of 5.9 nm for left panel and +80 mV with pore size of 5.5 nm for right panel. Insets : IV-curves and TEM images of the respective pores.

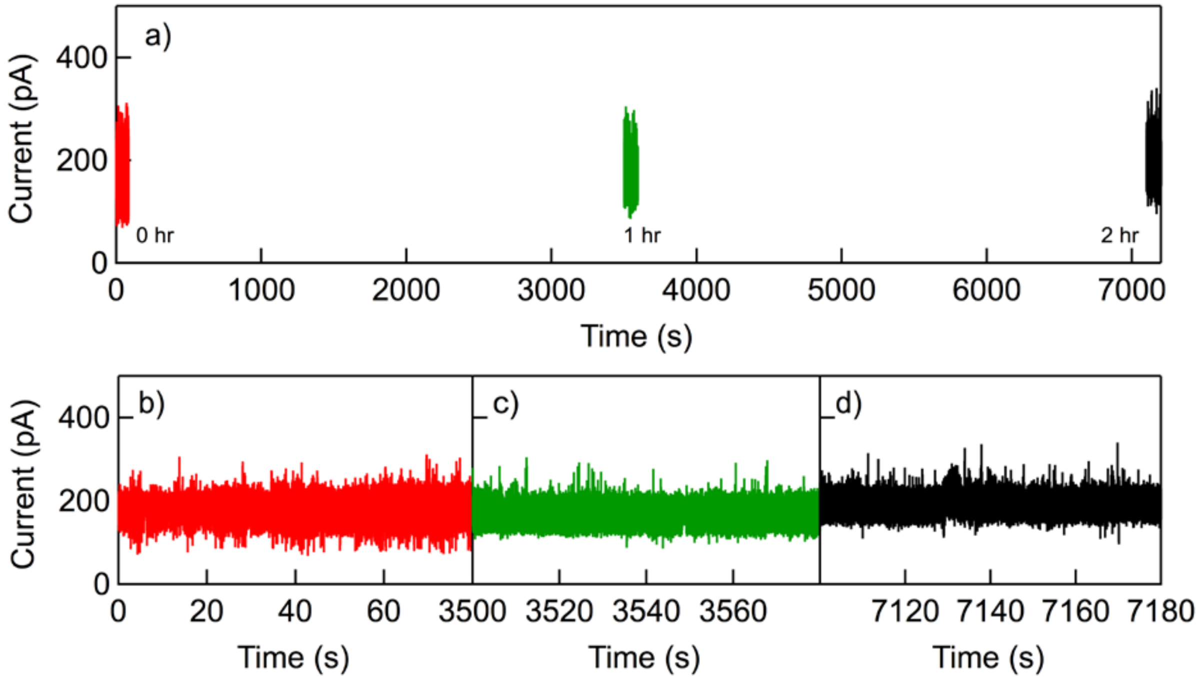

Supplementary Figure 4: Stability of the baseline ion current signal for a hybrid CGG portal over time. a) Ion current signals of a hybrid CGG portal, inserted into a 5.8 nm SS pore, at 100 mV recorded for 60s at hourly intervals. Traces were recorded at 0 (b, red), 1 (c, green) and 2 hours (d, black) after hybrid SS-portal nanopore formation. The slight increase in current observed at 2 hours (d, black) is likely due to evaporation as the cell was not sealed.

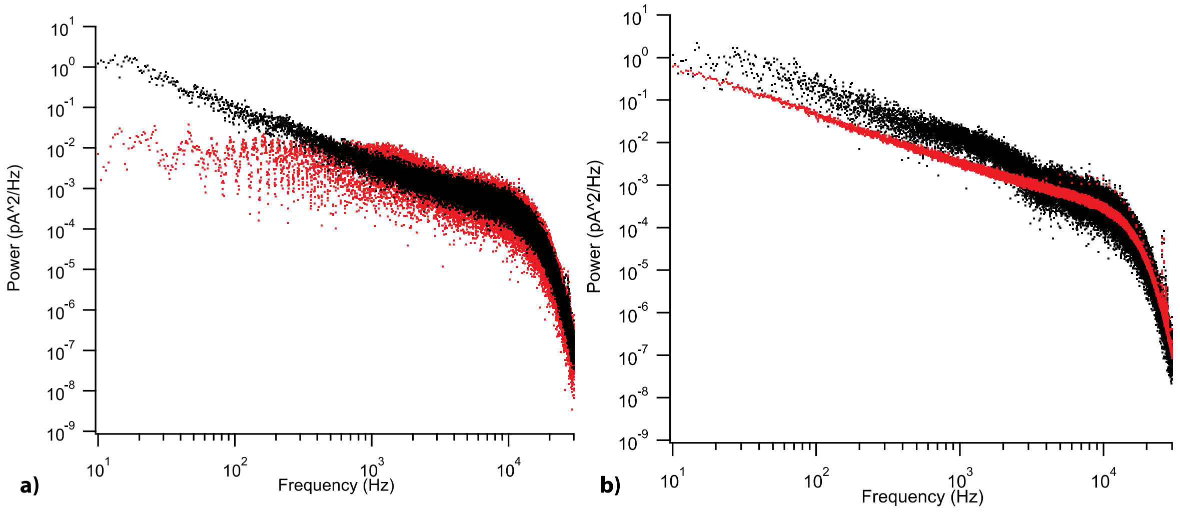

Supplementary Figure 5: Examples of typical current noise analysis. Traces show power spectral density of the current noise for a 5.4 nm (a) and 5.7 nm (b) diameter solid-state nanopore before (black) and after insertion of a portal protein (red) at +80 mV. Experiments were performed in 0.5 M NaCl, 20 mM Tris pH 7.5.

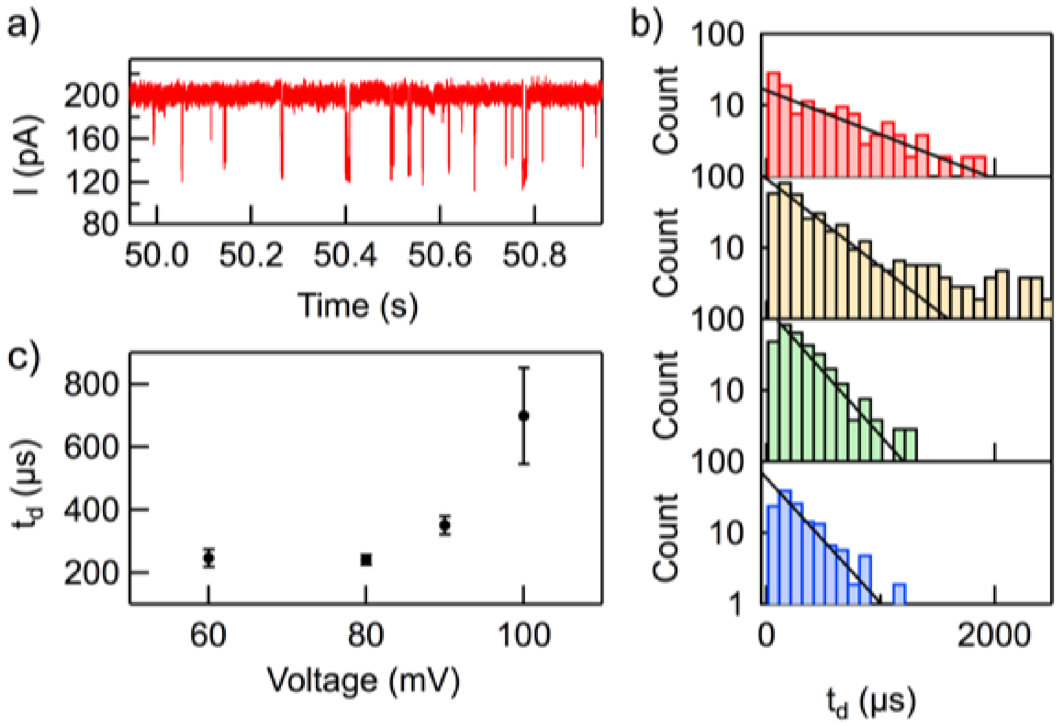

Supplementary Figure 6: Transport dynamics of β-cyclodextrin through a hybrid CGG portal protein. (a) hybrid pore current trace recorded at +100 mV after β-cyclodextrin addition to both chambers with total concentration of 0.3 mM. The β-cyclodextrin molecules are driven electro-osmotically to the CGG portal protein due to a K+ flow, in good agreement with the negative internal charges within the pore and extensive negative isocontours, as previously demonstrated[1]. (b) distribution of blockade duration for β-cyclodextrin at four different voltages: 100mV (red, n = 143 events), 90mV (yellow, n = 414 events), 80mV (green, n = 356 events), and 60mV (blue, n = 163 events). Black lines represent and exponential fit to the data. (c) β-cyclodextrin blockade duration as a function of voltage, error bars represent s.d. Data was collected from one hybrid nanopore.

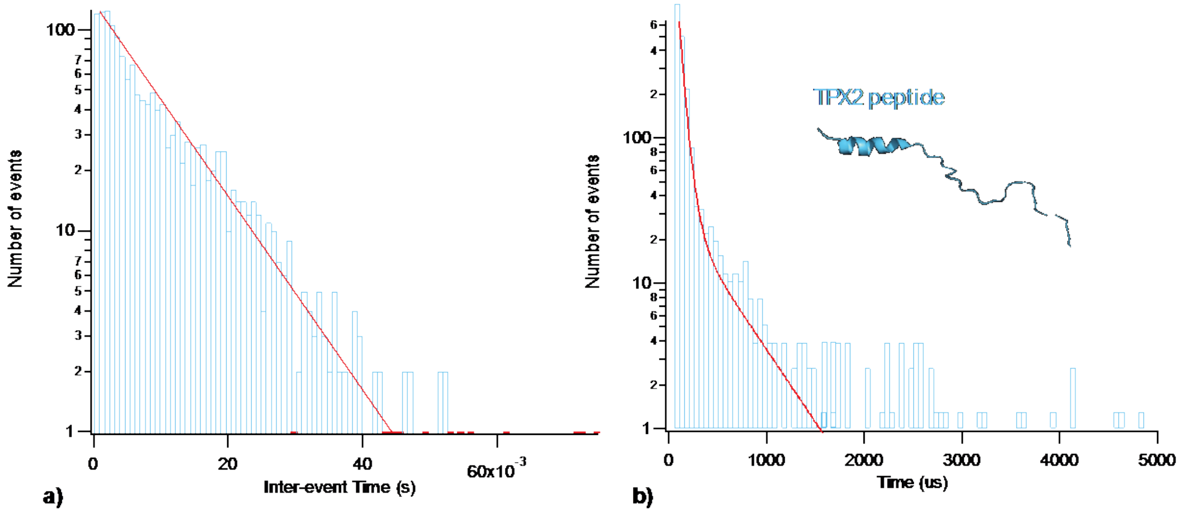

Supplementary Figure 7: Data analysis for frequency of events and dwell times. Distribution of duration between events for TPX2 at + 50 mV (a). The red line corresponds to an exponential fit. We find 120.5 $\pm$ 2.4 Hz. Distribution of blockade duration for TPX2 at + 50 mV (b). The red line solid line is a double exponential fit to. We find ι_1 = 35.2 $\pm$ 6.9 μs and ι_2 = 378.6 $\pm$ 84.1 μs. Data is calculated as the mean and s.d. of 1536 events from one hybrid nanopore.

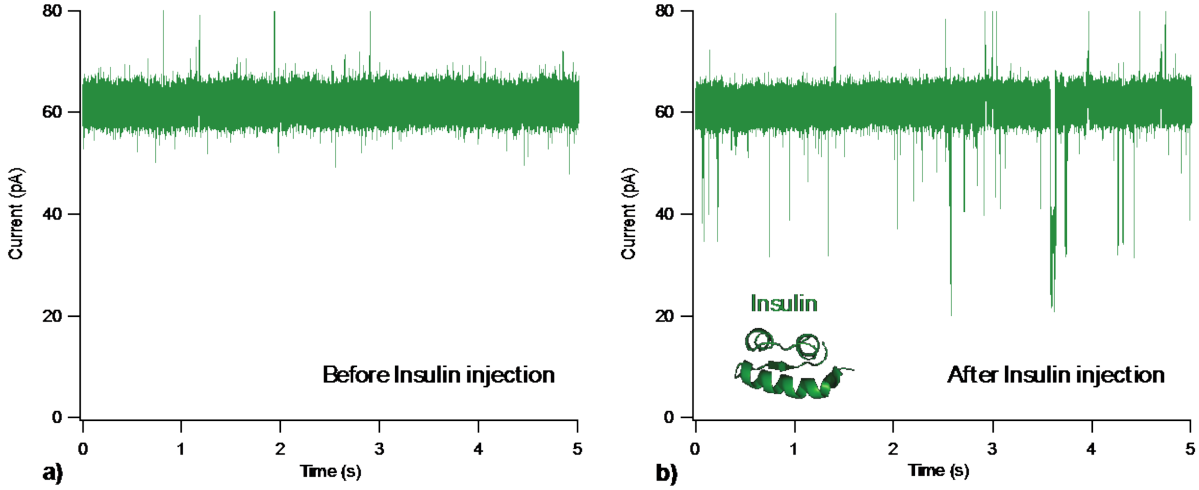

Supplementary Figure 8: Current traces of a portal hybrid pore before and after injection of insulin. Concentration of Insulin was 36 µM (b). Current traces were measured at (a, b) 60 mV.

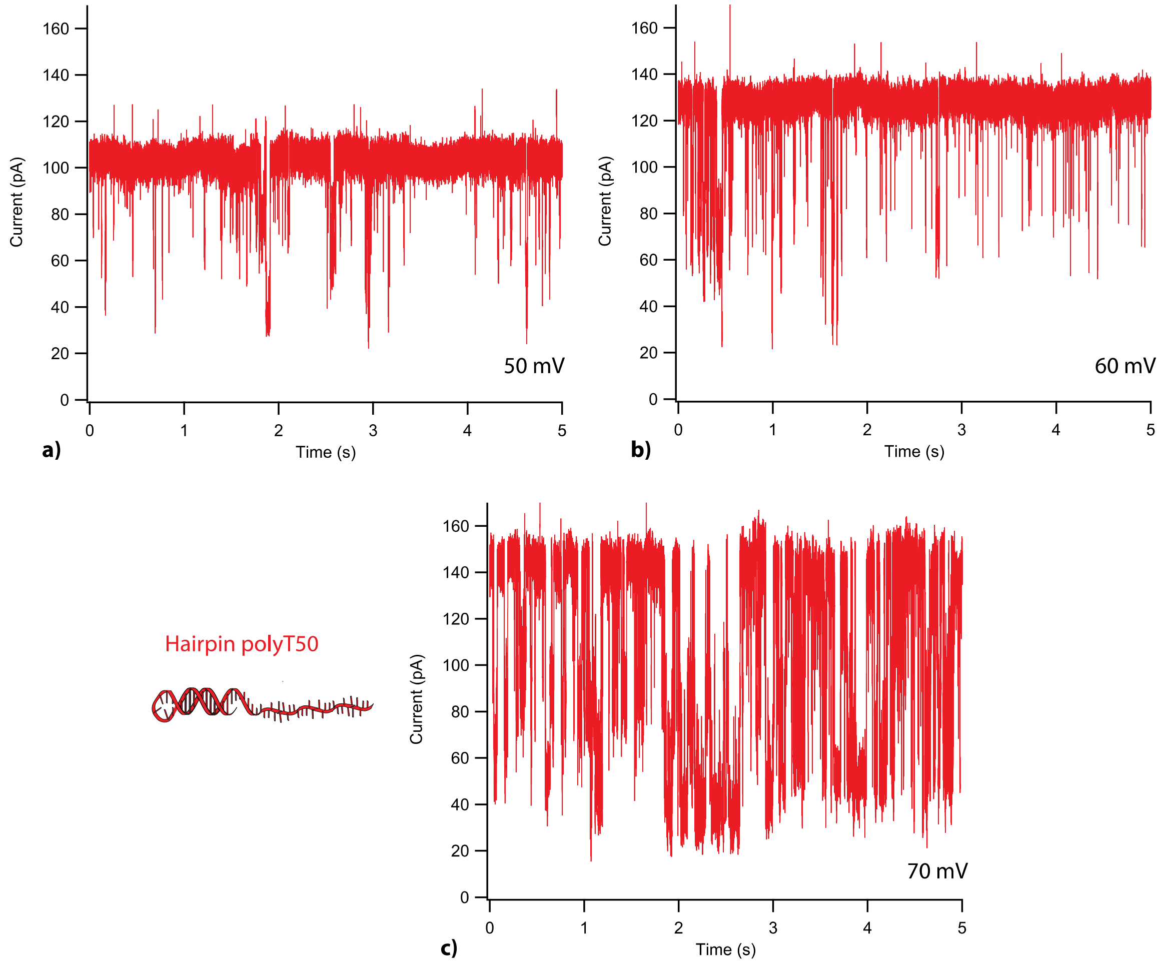

Supplementary Figure 9: Current traces of Hairpin polydT$_{50}$ transport through a portal hybrid pore at different applied voltages. Concentration of Hairpin polydT$_{50}$ was 10.3 µM. Current traces were measured at (a) 50 mV, (b) 60 mV, or (c) 70 mV.

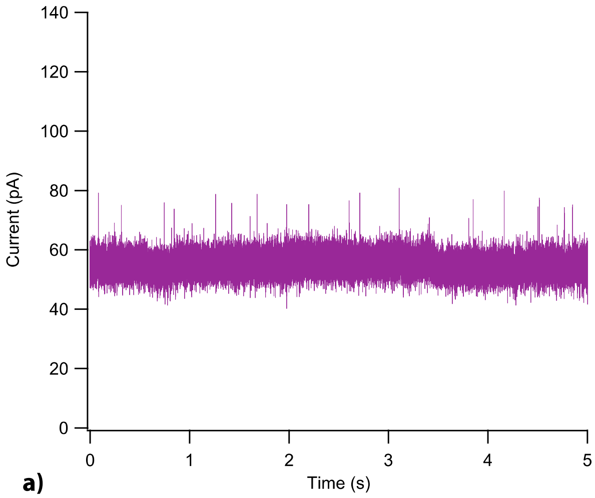

a)

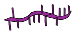

b)

c)

d)

Supplementary Figure 10: Current traces of a portal hybrid pore before and after injection of 60bp-ssDNA polydT$_{30}$ at different applied voltages. Concentration of 60bp-ssDNA polydT$_{30}$ was 6.9 µM (b, c, d). Current traces were measured at (a, b) 60 mV, (c) 80 mV, or (d) 100 mV.

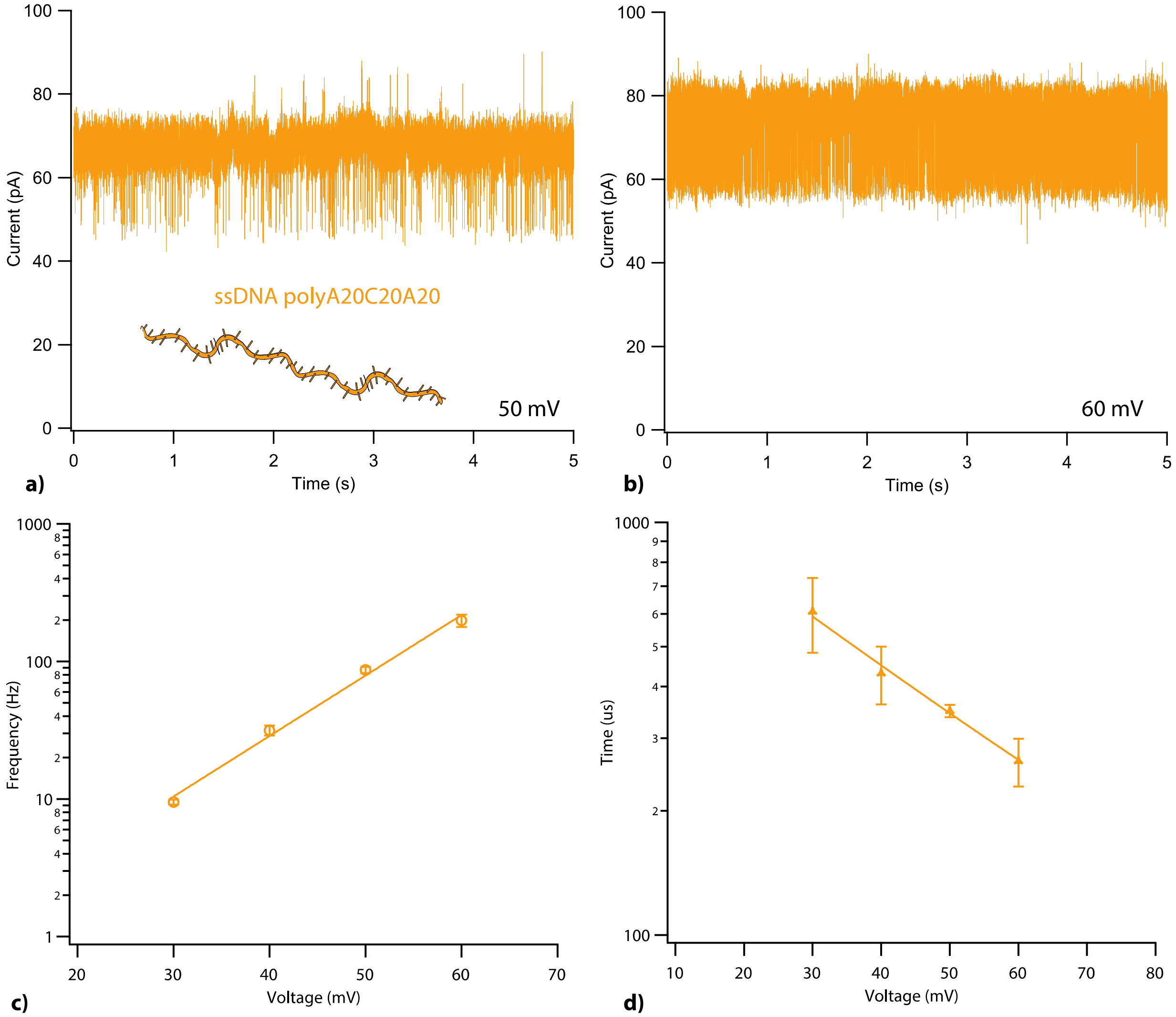

Supplementary Figure 11: Dynamics of ssDNA polydA$_{20}$dC$_{20}$dA$_{20}$ transport through a portal hybrid pore as a function of the applied voltage. Current traces of ssDNA polydA$_{20}$dC$_{20}$dA$_{20}$ transport through a portal hybrid pore at 50 mV (a) and at 60 mV (b). Frequency of events as a function of the applied voltage (c). Long current pore blockade times as a function of the applied voltage (d). Data shown in semi-log plots are mean and s.d. of 75,248 events (total for all points) from one hybrid nanopore. The line is an exponential fit.

Supplementary References:

1. Cressiot, B. *et al.* Porphyrin-Assisted Docking of a Thermophage Portal Protein into Lipid Bilayers: Nanopore Engineering and Characterization. *ACS Nano* **11,** 11931–11945 (2017).
2. Dolinsky, T. J., Nielsen, J. E., McCammon, J. A. & Baker, N. A. PDB2PQR: an automated pipeline for the setup of Poisson-Boltzmann electrostatics calculations. *Nucleic Acids Res* **32,** W665–7 (2004).
3. Baker, N. A., Sept, D., Joseph, S., Holst, M. J. & McCammon, J. A. Electrostatics of nanosystems: application to microtubules and the ribosome. *Proc. Natl. Acad. Sci. U.S.A.* **98,** 10037–10041 (2001).
4. Emsley, P. & Cowtan, K. Coot: model-building tools for molecular graphics. *Acta Crystallogr. D Biol. Crystallogr.* **60,** 2126–2132 (2004).
5. Tang, G. *et al.* EMAN2: an extensible image processing suite for electron microscopy. *J Struct Biol* **157,** 38–46 (2007).
